# Wearable and Noninvasive Device for Integral Congestive Heart Failure Management in the IoMT Paradigm

**DOI:** 10.3390/s23167055

**Published:** 2023-08-09

**Authors:** José L. Ausín, Javier Ramos, Antonio Lorido, Pedro Molina, J. Francisco Duque-Carrillo

**Affiliations:** 1 Department of Electrical, Electronics and Control Engineering, University of Extremadura, 06006 Badajoz, Spain; duque@unex.es; 2 BioBee Technologies S.L., Extremadura Science and Technology Park, 06006 Badajoz, Spain; javier.ramos@biobee.tech (J.R.); antonio.lorido@biobee.tech (A.L.); pedro.molina@biobee.tech (P.M.)

**Keywords:** congestive heart failure, cardiac output assessment, bioimpedance, CMOS integrated circuits

## Abstract

Noninvasive remote monitoring of hemodynamic variables is essential in optimizing treatment opportunities and predicting rehospitalization in patients with congestive heart failure. The objective of this study is to develop a wearable bioimpedance-based device, which can provide continuous measurement of cardiac output and stroke volume, as well as other physiological parameters for a greater prognosis and prevention of congestive heart failure. The bioimpedance system, which is based on a robust and cost-effective measuring principle, was implemented in a CMOS application specific integrated circuit, and operates as the analog front-end of the device, which has been provided with a radio-frequency section for wireless communication. The operating parameters of the proposed wearable device are remotely configured through a graphical user interface to measure the magnitude and the phase of complex impedances over a bandwidth of 1 kHz to 1 MHz. As a result of this study, a cardiac activity monitor was implemented, and its accuracy was evaluated in 33 patients with different heart diseases, ages, and genders. The proposed device was compared with a well-established technique such as Doppler echocardiography, and the results showed that the two instruments are clinically equivalent.

## 1. Introduction

Currently, new habits for healthy lifestyles are being accepted, many of them trying to implement preventive health programs and early detection of diseases, as the most effective way to improve the effectiveness of treatments and therapies and ensure, as far as possible, a high quality of life and healthy aging. These trends have led to the promotion of e-health and mobile health systems to improve healthcare services through the implementation of intelligent medical devices for personalized use, home/wearable and remote monitoring of physiological parameters [1].

The rapid advances in sensor-based systems and Internet technologies have enabled a new dimension of healthcare technology, namely Internet of Health Things or Internet of Medical Things (IoMT) [1,2]. IoMT is the exchange and processing of data through the Internet for health status monitoring of individuals by integrating smart sensors, advanced mobile technologies, and artificial intelligence, which brings great promise to the healthcare industry. IoMT can be broadly classified into two subcategories: personal and clinical. Personal IoMT includes devices such as activity/heart rate trackers and smartwatches that are used by consumers for self-monitoring without involvement from physicians. Clinical IoMT devices are built specifically for remote health monitoring of people with chronic or long-term conditions under the guidance and with the involvement of a physician. Examples include smart continuous glucose monitors and adhesive patch-type Holter monitors. These devices are intended for use in either clinical or home environments and are strictly regulated and approved for use after clinical validation. However, most of the pre-COVID-19 uses of IoMT were for small-scale application and in many cases when the cost and scalability were not an issue. Given the large-scale challenges caused by chronic diseases, very low-cost and effective wearable medical devices for IoMT have become of higher importance. This involves a wide range of technical challenges including accuracy, wearability, and ease-of-use (especially for the aged population) in unstructured dynamic environments. Furthermore, IoMT can be enhanced by artificial intelligence methods which endow IoMT systems with several degrees of intelligence [3].

Chronic cardiovascular diseases are the leading cause of morbidity and mortality in patients over 65 years of age, being one of the most important challenges facing current healthcare systems [4]. Cardiovascular disease is a general term for conditions affecting the heart or blood vessels, which includes all heart and circulatory diseases, such as congestive heart failure (CHF). Patients with CHF present with a variety of symptoms, most of which are non-specific, and some symptoms of CHF may be like geriatric diseases, which can lead to delays in treatment. Diagnosis of CHF is usually done after reporting the first symptoms to general practitioners, who may refer the patient to the cardiologist for complete evaluation. The assessment of the function of the heart by the cardiologist is made by monitoring stroke volume (SV)—volume of blood pumped per beat by the heart—and cardiac output (CO)—average of SVs per minute—which are usually reduced in patients with CHF. The Swan–Ganz catheter method has been traditionally used because of its precision, but due to its invasive nature (insertion of catheter through pulmonary artery), it is not exempt from complications and is only utilized in very specific cases. As a noninvasive method, one of the most-used methods for critical patient hemodynamic assessment is the Doppler echocardiography, which has complemented the use of the Swan–Ganz catheter in most Intensive Care Units and Coronary Units. However, Doppler is operator-dependent and requires a learning curve, making it not convenient for routine practice.

Once the patient is diagnosed, specific treatment is given, and periodic ambulatory monitoring checks are made to control heart function. Despite these ambulatory controls, there is a high rate (44%) of readmissions, as the heart behavior is not controlled continuously. In the next disease stages, people might need more support and hospitalizations. There can be ups and downs, with a general worsening of the disease and symptoms over time. These periods are associated with an increase in mortality and morbidity following discharge and are responsible for most of the costs related to CHF care. Additionally, due to the lack of appropriate means outside cardiology units, CHF cases are usually not detected soon enough. Detecting CHF in early phases is difficult because symptoms and signs such as fatigue, shortness of breath, and edemas are not specific, and obvious symptoms are present only when the disease becomes severe.

Therefore, early prediction of CHF is considered crucial in order to diagnose it as soon as possible to minimize the risk [5], and automatic detection through clinical IoMT devices, which enable noninvasive and continuous hemodynamic monitoring, plays a significant role in managing the large number of patients suffering from CHF [6]. Promising alternative techniques for noninvasive and continuous monitoring of cardiac activity include variations of electrical bioimpedance magnitude—impedance cardiography (ICG) or thoracic electrical bioimpedance (TEB)—and bioimpedance phase in the thorax (bioreactance) originated by the amount of pulsatile blood flow in the aorta [7]. However, they are only applicable in an in-hospital environment as bedside equipment and, hence, the bulky and non-wearable form factor makes it impossible to be used in an IoMT scenario.

### Scope and Purpose of the Presented Work

As a result, a key objective of this work is to design a bioimpedance measurement sensor that can be integrated into a multi-modal and wearable device, which combines both continuous hemodynamic assessment and physiological parameters related to breath behavior, pulmonary edema, and muscular physiology measuring capabilities. The proposed wearable device will power Primary Care to have a vital role in identifying persons with CHF and in providing holistic, person-centered care from the first symptoms to end of life, as the 2021 European Society of Cardiology Guidelines for the diagnosis and treatment of acute and chronic heart failure consider (Class IIb recommendation) [4].

The rest of the paper is organized as follows. Section 2 outlines the fundamentals of bioimpedance technology, explaining how it is applied to evaluate cardiac function, and gives a review of noninvasive bioimpedance-based monitoring devices for CHF on the market. The designed bioimpedance sensor, which was realized as a CMOS application specific integrated circuit, and the rest of the building blocks of the proposed multi-modal and wearable device are described in Section 3. The experimental performance of the sensor and the wireless device is provided in Section 4. As a target IoMT application, Section 4 also deals with the realization of a continuous cardiac output monitor based on the proposed device and its analytical/clinical validation in the adult study population. Finally, the findings are discussed in Section 5, and conclusions are drawn in Section 6.

## 2. Electrical Bioimpedance Technology

In the context of healthcare, electrical bioimpedance or, simply, bioimpedance, refers to the analysis of voltage signals in the response of a human body to an applied low-level alternating current [8,9,10]. Bioimpedance (*Z*) is defined as a complex ratio of a recorded voltage (*V*), appearing across a pair of electrodes, over an alternating current excitation signal (*I*), i.e., *Z* = *V*/*I*, through these electrodes and, hence, represents the opposition of a conductor to the circulation of an electric current [9]. Then, bioimpedance is calculated as

(1) $$Z\left(j\omega \right)=\frac{V\left(j\omega \right)}{I\left(j\omega \right)}=\frac{V_{o}\cdot e^{j\phi }}{I_{o}}=\left|Z\right|\cdot e^{j\phi }=Re\left(Z\right)+jIm\left(Z\right)$$

where *I_o_* and *V_o_* are the injected current and the recorded voltage amplitudes, respectively, *ω* = 2π*f* is the test signal angular frequency, and *ϕ* is the phase difference between current and voltage [10]. It is evident that bioimpedance can be described either by a magnitude and phase, i.e., |*Z*| and *ϕ*, or equivalently by the real and imaginary parts of the impedance. As a result, bioimpedance is a function of the tissue structure and composition as well as of the frequency of the excitation current signal. 

**Figure 1:**
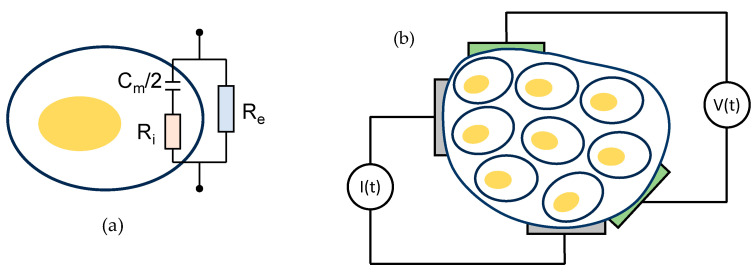
(**a**) Simplified equivalent circuit (2R1C) of a single cell in the extracellular medium; (**b**) four-electrode bioimpedance measurement technique.

A biological tissue can be understood as a group of similar cells and associated intracellular matter acting together to perform specific functions in an organism. In healthy tissues, the cell membrane acts as a boundary between the intracellular components and the extracellular medium. The membrane allows lipids and water molecules to pass through it but, in principle, acts as a barrier for ions. From the electrical behavior point of view, the cell membrane can be considered as a dielectric material and, hence, the structure formed by the extracellular medium, the membrane, and the intracellular components behaves as a capacitor, C_m_ [11]. The current signal injected into the extracellular medium can flow in the cell through C_m_, through the ionic channels, or can also circulate around the cell. Since the ionic channels and the extracellular medium behave as a resistor and the membrane conductance is extremely low, the equivalent circuit of a single cell in the extracellular medium consists of two resistors and one capacitor (2R1C). This is illustrated in Figure 1a, where R_i_ represents the intracellular matter resistance and R_e_ represents the extracellular fluid resistance. The same equivalent circuit can be used to represent a tissue composed of many cells. This circuit is adopted by most authors and explains the bioimpedance behavior from a few hundreds of Hz to some tens of MHz, which is known as β-dispersion range [8]. At frequencies below 1 kHz, the most significant pathways are found in the extracellular fluids because the current is not able to penetrate the cells. At frequencies higher than 1 MHz, the current penetrates the cell membranes and flows through both the intra- and the extracellular environments. As a result, the bioimpedance remains relatively constant regardless of physiological behaviors. For that reason, typical frequencies of electrical signals used in bioimpedance technology for medical diagnosis purposes range from 1 kHz to 1 MHz. The amplitude of the excitation current signal is chosen with such low intensity (in range of µA and always lower than a few mA) that bioimpedance, besides noninvasive and easy-to-use, remains innocuous.

Electrodes are always necessary to measure bioimpedance in order to establish proper electrical connections with the biological material. The arrangement and quantity of electrodes used in a bioimpedance measurement significantly affect the results obtained [9]. Various techniques utilize two, three, or four electrodes. However, in this study, as usual, the four-electrode (tetrapolar) configuration (Figure 1b) is employed because it is less affected by changes in the electrode/tissue impedance of both the voltage-sensing and current-injecting electrodes [10].

In practice, there are two different types of analysis in bioimpedance technology. One of them consists of using a sinusoidal excitation current signal with a single frequency (SF) component. This is known as bioelectrical impedance analysis [9]. However, for characterizing certain alterations in the composition or detecting some transient physiological events, SF-bioimpedance cannot be adequate. In such cases, bioimpedance spectroscopy (BIS) is used. BIS implies the measurement of the impedance spectrum in a determined frequency range, for which a sequential sweep of analysis varying the signal frequency is carried out. However, the use of traditional BIS results are unsuitable in applications where either the detection and characterization of fast physiological transient events are required, or the availability of measured bioimpedance parameters must be immediate. Also, even though conceptually there is no difference, some authors distinguish between BIS and multi-frequency bioimpedance analysis. The only difference resides in the number of frequencies contained in the sweep and, although the separation limit is not very clear, the number of frequencies in a BIS assay is larger than in its multi-frequency bioimpedance counterpart. Logarithmic or quasi-logarithm frequency distributions are often preferred and can be found in commercial BIS equipment. Other methods have been proposed to obtain a frequency distribution that improves the results of BIS measurements within the β-dispersion range using a limited number of frequencies [12].

**Figure 2:**
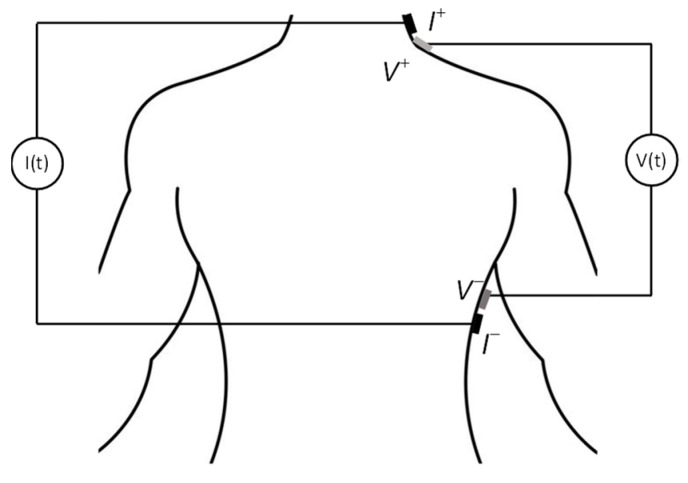
Standard ICG four-electrode measurement setup.

Bioimpedance technology has been applied for (1) dynamic monitoring of physiological transient events (respiration, hemodynamic/cardiac parameters), (2) slow evolving parameters, e.g., muscular alterations and fluid accumulation detection, and (3) medical imaging of a specific region in the human body (electrical impedance tomography). SF-bioimpedance is usually applied to assess dynamic events or muscular alterations. Multi-frequency bioimpedance and BIS are used in the rest of the mentioned applications. One of the implementations of SF-bioimpedance, ICG, is widely used as a non-invasive procedure for monitoring hemodynamic parameters such as SV and CO [13]. Concretely, CO is given by the product of SV and heart rate (HR), i.e., CO (mL/min) = SV (mL/beat) × HR (beat/min). ICG is performed by using a constant excitation current and a four-electrode arrangement. Usually, current signals within the frequency range of 20–100 kHz are employed to excite the driving electrodes. As shown in Figure 2, the typical setup involves placing the driving electrodes around the abdomen and the upper part of the neck. The sensing electrodes are then positioned around the thorax and the lower part of the neck.

This arrangement allows for the current to flow in the direction of the blood flow [13]. There are numerous equations implemented by the ICG approach, but the most common formula is the one stablished by Kubicek and his coworkers [14,15]:

(2) $$SV=\rho \cdot {\left(\frac{L}{Z_{0}}\right)}^{2}\cdot {\left(\frac{dZ}{dt}\right)}_{max}\cdot T_{LVE}$$

where *ρ* is the blood resistivity, which is generally taken as a constant value 135 Ω.cm, *L* denotes the thoracic length between voltage electrodes in cm and is approximated as 17% of the patient’s height, *Z*_0_ is the measured mean basal impedance in Ω, (*dZ*/*dt*)_max_ corresponds to the maximum rate of a change in bioimpedance, and *T_LVE_* is the left ventricular ejection time. Sramek, Bernstein, and Osypka introduced additional formulas, as did the equipment producers. However, it remains challenging to find a formula for SV calculations that consistently provides accurate results, regardless of the patient’s condition [14]. Recently, it has been shown that simultaneous recording of both SV and a signal that reflects pulmonary congestion can provide additional diagnostic data for predicting CHF. BIS has demonstrated the ability to detect and follow the initial stages of pulmonary congestion, which are characterized by small volumes of excess fluid contained in the pulmonary vasculature [16].

In the recent past, multidisciplinary approaches for obtaining telemonitoring information via noninvasive bioimpedance-based devices have been studied and validated for different pathologies [17,18]. Undoubtedly, they are very promising, both for costs and for noninvasiveness, and would make person-centered care feasible in patients with a high risk for complications. Nevertheless, bioimpedance technology suffers from some disadvantages, mainly deriving from the fact that it does not directly measure any physiological parameter. Therefore, calibration of the bioimpedance in physiological terms is required. This has limited its presence in the clinical device market. Moreover, the data obtained by bioimpedance are not usually interchangeable with those obtained from other methods, e.g., echocardiographic method in hemodynamic evaluation [19]. However, these drawbacks are avoided when bioimpedance is used to monitor the progression of the disease in critical patients [20] and helps physicians to create personalized healthcare plans. This is the solution adopted in this work.

To the authors’ knowledge, although noninvasive and efficient patch-based wearable monitoring for early detection of CHF is not yet available, several commercial approaches exist, and others are on the way [21]. In particular, two wearable approaches are on the market: CoVa Necklace [22] and µCor3 [23]. The CoVa system is a wearable necklace for home monitoring worn by patients for at least 5 min a day. The necklace has been designed following the classical bioimpedance measurement principle. The small size has been obtained by simplifying capacities, so their use is limited in frequency, suffering from a loss of information in other frequencies (being the loss of information crucial for an appropriate early detection of CHF). Concretely, it operates with a unique frequency of 70 kHz. µCor3 is a wearable adhesive chest patch designed for heart failure patients with fluid management problems. It continuously records patient data including Thoracic Fluid Index, heart and respiration rates, activity, posture, and heart rhythm (ECG). It is based on radiofrequency (RF) bioimpedance techniques and does not provide information about either the parameter related to cardiac output nor respiration volumes. Finally, it is worth it to mention a Holter-size wireless cardiac output monitor, although not designed for CHF monitoring but for ICG, that provides important cardiac parameters (SV/CO, heart rate, and respiration flow) while a subject or patient is exercising [24].

## 3. Materials and Methods

### 3.1. Multi-Modal and Wearable Bioimpedance-Based Sensing Device

**Figure 3:**
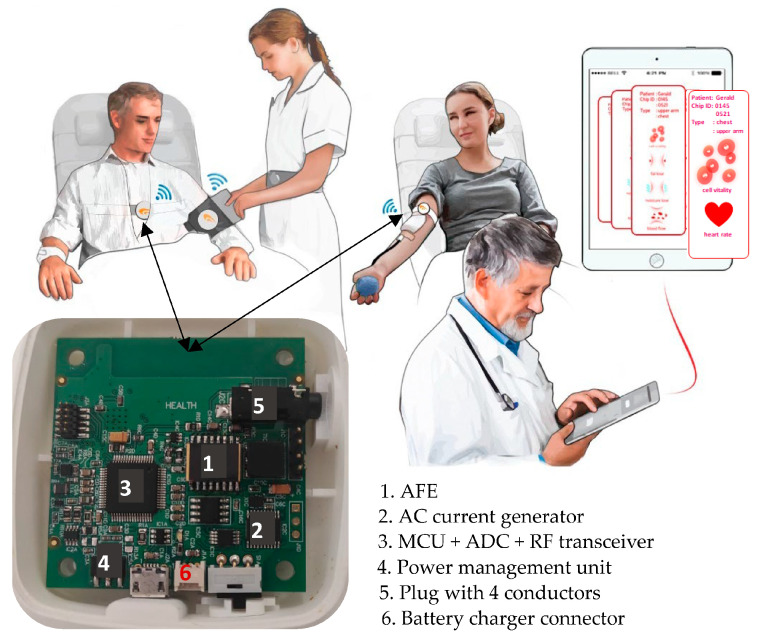
System overview of the developed bioimpedance device for continuous hemodynamic assessment and physiological parameters related to breath behavior, pulmonary edema, and muscle quality.

Figure 3 shows a picture of the developed sensing device as well as its intended functionality. The size of the device is 6 cm × 6 cm, including the printed circuit board, the battery, and the wireless antenna. For improved comfortability, the device utilizes a rigid printed circuit board in a customized waterproof enclosure that allows to be used heavily day after day and still look as new as possible. The proposed bioimpedance-based sensing device consists of four major functional units: (1) a custom-made CMOS analog front-end (AFE) integrated circuit for bioimpedance sensor, (2) a digitally programmable alternating current (AC) generator, (3) a low-power microcontroller unit (MCU) with integrated analog-to-digital converter (ADC) and RF transceiver section, and (4) power management unit. The frequency of bioimpedance measurements can be set from 1 kHz to 1 MHz by changing the control bits in the MCU according to the command received by the transceiver. The recorded bioimpedance data can then be wirelessly transmitted to an IoT gateway that forwards the data to the cloud storage to apply analytic techniques, e.g., machine learning, to generate predictions or decision support to help healthcare professionals involved in the diagnostic process.

### 3.2. Bioimpedance Sensor (AFE)

The most popular and well-established measuring technique used in monolithic implementations of bioimpedance sensors is based on the analog quadrature demodulation (AQD) method [9,10,25]. Using this technique, the extracted outputs are two direct current (DC) voltage signals proportional to the real and the imaginary part, respectively, of the bioimpedance under test. However, the AQD method is very sensitive to unavoidable mismatches between the in-phase and the quadrature channel, which causes phase errors in the corresponding in-phase and quadrature reference signals and, hence, errors in the DC output voltage levels that represent the real and the imaginary part of the measured bioimpedance. In addition, these errors are greater when the frequency of the signal used is increased, which is unacceptable for BIS applications. Hence, a correction circuit is needed, increasing the circuit complexity [26]. Digital quadrature demodulation (DQD) is, in principle, an alternative method for avoiding phase errors coming from mismatches in AQD measurements. However, DQD requires digital demodulators based on digital signal processors or field programmable gate arrays and, hence, adds such a complexity to the whole measurement circuit realization that it often leads to high cost, high size, and high power consumption implementations. Other demodulation methods have been reported in the literature. In [27], a time-to-digital method for bioimpedance implant applications was proposed. It merges the readout circuit with digitization instead of using a separate ADC, resulting in a simple and low power design. The proposal in [28] suggests using lock-in amplifiers to convert impedance to frequency in wearable systems. The lock-in amplifiers extract the real and imaginary parts of the impedance. 

**Figure 4:**
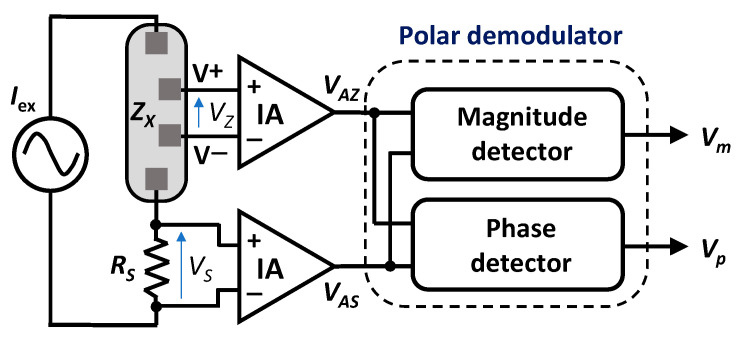
Simplified block diagram of a polar demodulator architecture.

Polar demodulation is a promising alternative which asynchronously measures the magnitude and phase of the bioimpedance [29,30,31,32,33,34], as shown in Figure 4. It is a tetra-polar measurement technique based on determining the magnitude and the phase of a complex impedance, *Z_X_*, by extracting and processing two voltage signals, *V_AZ_* and *V_AS_*, obtained as a response to a sinusoidal excitation current, *I*_ex_. As shown in Figure 4, the sinusoidal excitation current is injected into the biological tissue through the first outer electrode. This current flows through the biological sample portion, which is delimited by two (inner) sensing electrodes, V+ and V–, and a passive reference resistor, *R_S_*. The excitation current flowing through *Z_X_* and *R_S_* gives rise to voltages *V_Z_* and *V_S_*, respectively. These two signals are amplified by two instrumentation amplifiers (IAs) and, thus, yield two voltage signals, *V_AZ_* and *V_AS_*, which are then compared to obtain two DC voltages, *V_m_* and *V_p_*, proportional to the magnitude and phase of *Z_X_*. In general, this alternative constitutes a compact and cost-effective indirect technique for real-time bioimpedance measurements and features outstanding advantages over traditional techniques based on AQD. 

There are various approaches to measure the magnitude and phase of a complex impedance with the polar demodulator architecture. In [29], a portable BIS device based on the magnitude-ratio and phase-difference detection (MRPDD) method was presented. The device was implemented in discrete form with commercial analog electronics circuits. In particular, a gain-phase detector AD8302 from Analog Devices was used. Since the AD8302 chip is intended for communication applications up to 2.7 GHz, a large power consumption is required [29,30]. A custom integrated circuit (IC) architecture that relies on a self-mixing full-wave rectifier was proposed in [31]. This solution eliminates IA offset errors not addressed in [29,30]. Two adaptive self-sampling schemes are proposed in [32]. These schemes, which do not have issues related to synchronization and settling time, are designed to efficiently measure the magnitude in BIS measurements. In [33], a frequency-shift technique is presented. This technique aims to reduce the demodulator operation frequency and the overall IC power consumption.

**Figure 5:**
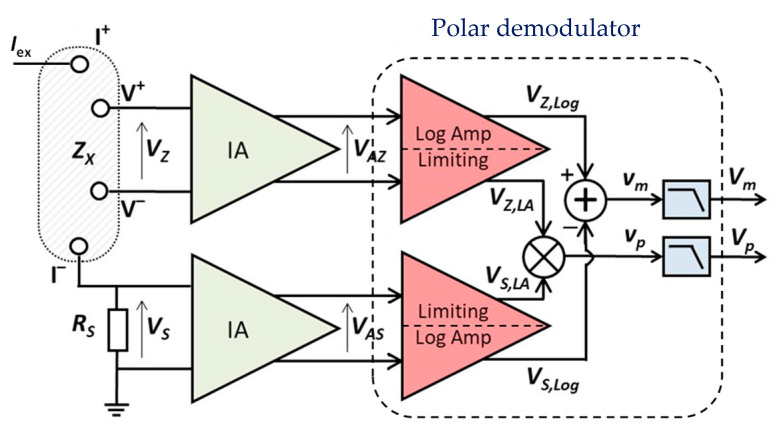
Simplified block diagram of the proposed analog front-end.

The monolithic CMOS realization of the bioimpedance sensor employed in this work is based on the MRPDD method [34]. As shown in Figure 5, the sensor uses two logarithmic amplifiers to measure the magnitude. For phase measurement, limiting amplifiers are used to convert *V_AZ_* and *V_AS_* into two square-wave signals, *V_Z_*_,*LA*_ and *V_S_*_,*LA*_, that are further compared by a simple XNOR gate. Therefore, after low pass filtering, two DC voltages, *V_m_* and *V_p_*, proportional to their magnitude ratio $\left|K\right|$ and phase difference *θ*, respectively:

(3) $$\left|K\right|=\left|\frac{V_{AZ}}{V_{AS}}\right|$$

*θ* = *θ_AZ_* − *θ_AS_* (4)

where *θ_AZ_* and *θ_AS_* are the phases of signals *V_AZ_* and *V_AS_*, respectively. Assuming the two measurement channels to be identical, the impedance under measurement is obtained as

(5) $$Z_{X}=R_{S}\cdot {\left|K\right|}_{∠\theta }$$

The polar demodulator, which corresponds to the circuit portion of Figure 4 enclosed by the dashed line, consists of two matched limiting/logarithmic amplifiers (LLAs), a multiplier, and a subtractor. Each LLA is a non-linear amplifier with two output voltages [35], namely, a pseudo-logarithmic output, *V_Log_*, which represents a (pseudo) logarithmic conversion of the corresponding input signal, and a limited output, *V_LA_*, which varies linearly with the input voltage (linear operation) for sufficiently low input levels and is a saturated signal for higher input levels, which represent most of the amplifier input dynamic range. To achieve the necessary input dynamic range, the LLA is implemented with a successive-detection architecture, specifically using a parallel-summation technique [36].

Taking advantage of the arithmetic of logarithms, the difference between the two logarithmic output signals, *V_Z_*_,*Log*_ and *V_S_*_,*Log*_, is carried out, and the resultant signal *v_m_* (output of the subtractor) is low-pass filtered to obtain the DC component *V_m_* proportional to the desired magnitude ratio $\left|K\right|$ of signals *V_AZ_* and *V_AS_*. In the proposed AFE, the subtraction required to obtain *V_m_* is carried out digitally by the microcontroller unit that is also used to control the whole system operation. In this respect, it is worth it to point out that first performing the subtraction *V_Z_*_,*Log*_ − *V_S,Log_* and then filtering the obtained difference gives the same result as when first *V_Z_*_,*Log*_ and *V_S_*_,*Log*_ are low-pass filtered and then, the difference between the obtained DC signals is performed. Thus, in our implementation, the logarithmic outputs, *V_Z_*_,*Log*_ and *V_S_*_,*Log*_, of the two LLAs are filtered to extract their DC components, which are then converted to the digital domain by the ADC shown in the system overview of Figure 3, and finally subtracted by the MCU, thus producing the desired magnitude ratio signal *V_m_*.

**Figure 6:**
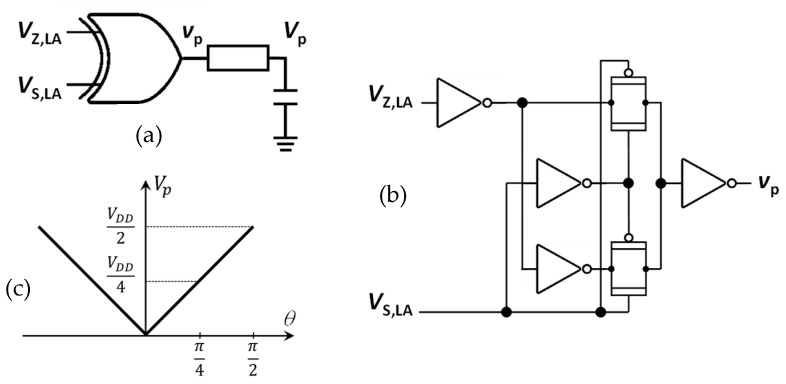
(**a**) XOR logic gate used in the phase measuring circuit; (**b**) its transistor-level implementation, and (**c**) the DC component *V_p_* of the pulse train *v_p_*.

The phase relationship between *V_AZ_* and *V_AS_* is derived from the limited output signals, *V_Z_*_,*LA*_ and *V_S_*_,*LA*_*,* of the two LLAs, which are almost perfect hard-limited square waves. An XOR gate, which is represented as a multiplier in Figure 5, processes the two limited outputs to generate a pulse-width modulated waveform of varying duty cycle, *v_p_*, whose average duty cycle represents the phase difference of the limited square-wave signals *V_Z_*_,*LA*_ and *V_S_*_,*LA*_, which, in turn, is the same as the phase difference of input signals *V_Z_* and V_S_. The value of the average duty cycle is then obtained by extracting the DC component of the pulse train waveform by means of a low-pass filter, as shown in Figure 6. Finally, the obtained phase difference signal *V_p_* is converted to the digital domain (ADC in Figure 3) and then fed to the MCU. When the phase difference of *V_Z_* and *V_S_* is *θ*, *V_p_* is expressed as:

(6) $$V_{p}=V_{DD}\cdot \frac{\theta }{\pi }$$

Classical phase extractor circuits, such as XOR gates or two edge-triggered flip-flops, present errors when the input waveforms are distorted by offset voltages introduced by preceding analog stages, e.g., limiting amplifiers. Digital waveforms will change state either slightly before or slightly after (depending on the polarity of the offset error) the instant when the associated input signal crosses its zero level. This leads to a timing error in the digital waveforms and, hence, results in a loss of accuracy in the phase measurement. In the proposed AFE, accuracy is guaranteed by employing a feedback-type offset cancellation scheme in limiting amplifiers. A low-pass transfer function filters the outputvoltage-limited signals and extracts the DC offset voltage, which is then subtracted from the signal at its input. This way, the input-referred offset voltage of the limiting amplifier is reduced by the closed-loop feedback network. Therefore, our proposed AFE becomes more immune to phase measurement errors caused by delay mismatches.

It is worth it to point out that the magnitude and phase values of the unknown impedance *Z_X_* are obtained by comparing voltage signals related to the same injected current *I*_ex_. A precise knowledge of the value of this current is not required, unlike in traditional measurement approaches based on the AQD method. This feature is especially important in the case of high-frequency BIS, where an increasing voltage drop across the output resistance of the excitation current source with increasing operating frequency unavoidably takes place, thus resulting in variations in the injected current. In addition, magnitude/phase measurement with the MRPDD method is the simplest and most straightforward technique able to provide the magnitude and phase information encoded in a pair of DC voltage signals, namely, *V_m_* and *V_p_*. The above characteristics simplify the complexity and the requirements of the hardware and the software necessary for bioimpedance signal processing and, hence, allow implementing a high-performance wireless bioimpedance analyzer/spectrometer on a board with reduced dimensions.

### 3.3. Wireless Bioimpedance Device

The core of the wireless bioimpedance sensing device is an ultra-low power microcontroller unit of Texas Instruments (TI)’s family CC26xx [37]. In particular, the MCU chosen—CC2650—provides a compact solution for the intended application. This circuit is equipped with two serial interface modules, which can support synchronous communication protocols such as serial peripheral interface. In addition, the inter-integrated circuit interface (I2C) is used to communicate with slow-speed peripheral ICs. In particular, the MCU controls the excitation current generator of the bioimpedance sensing device (see Figure 3), which is an Analog Devices (AD)’s AD9834 IC—Direct Digital Synthesizer—widely used in low-power medical equipment and RF systems. The MCU controls the frequency of the generated current signal by means of the serial peripheral interface, while the current signal amplitude is set by means of a digital potentiometer controlled through the I2C bus (AD’s AD5241). The programmability ranges of the excitation current in terms of signal amplitude and frequency are 5 µA to 1 mA and 1 kHz to 1 MHz, respectively.

To optimize the measurement operating range, an off-chip resistor was connected in parallel with on-chip reference resistor *R_S_* through a digital switch that is controlled via the I2C interface of the MCU. A tuning routine selects the adequate value of the excitation current amplitude and the total reference resistor to adapt the measurement range to the value of the unknown impedance *Z_X_* at any moment.

The analog-to-digital conversion required for signal *V_p_* and the low-pass filtered logarithmic outputs of the two LLAs (see Section 3.1), which carry the phase and the magnitude information, respectively, of the biological sample impedance, are digitized by means of a low-power 12-bit (10-bit ENOB) ADC, 200-ksamples/s, integrated in the CC2650 (SINAD = 63 dB). The selected MCU incorporates an ARM Cortex-M0 processor that interfaces the analog RF and base-band circuitries, handles data to and from the system side, and assembles the information bits in a given packet structure. The RF core targets Bluetooth Low Energy (BLE), ZigBee^®^ and 6LoWPAN, and ZigBee RF4CE remote control applications. In particular, the bioimpedance device operates with BLE standard, which is a low-power protocol fully incorporated in current smart mobile devices, that provides a high connectivity level while still allowing a very compact solution.

Finally, in order to obtain an efficient performance of the whole system, a power management microcontroller routine that optimizes power consumption during the active time of the measuring tasks was developed. In addition, the system enters an idle state during inactive (i.e., no measuring) time.

## 4. Results

### 4.1. AFE Experimental Performance

The AFE was designed to operate with 2 V of supply voltage and fabricated in standard 0.35-µm CMOS technology. Measurements on several loads of known impedance connected between the two sensing electrodes (V^+^, V^−^—see Figure 4 and Figure 5) were carried out to characterize the behavior of the AFE. 1%-accuracy metal film resistors and 5%-accuracy ceramic capacitors were used for these measurements. The electrode–skin interface present in actual bioimpedance measurements was emulated by means of the series connection of a 50 Ω-resistor, *R_es_*, and a 100 nF-capacitor, *C_es_* [8]. Considering the very high value of the input impedance of the AFE, only the electrode–skin interface of the two outer electrodes (I^+^, I^–^) was taken into account for these measurements—in practice, an *R_es_*-*C_es_* group was placed in series between each outer electrode and the corresponding terminal of the load to be measured, whereas the two inner electrodes (V^+^, V^−^) were directly connected to the load. Moreover, the IAs designed in this work have a bandpass-type transfer function in order to filter out low-frequency noise and electrode-induced drifts.

**Figure 7:**
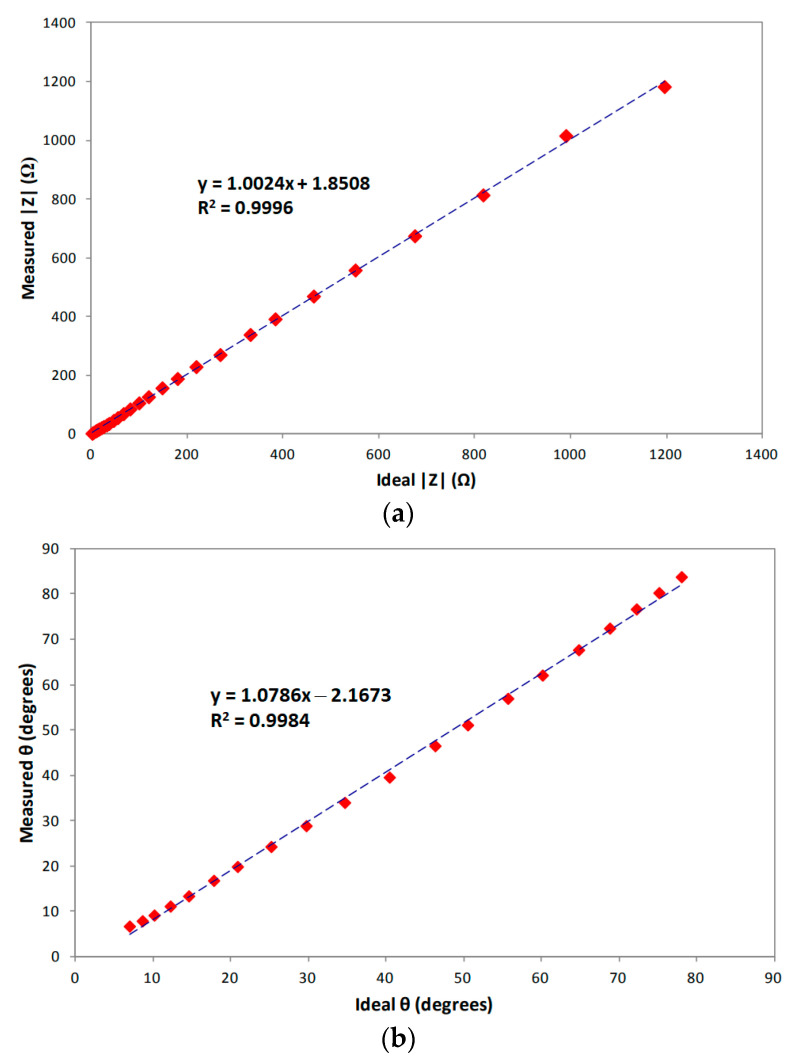
Experimental characterization of the CMOS AFE: (**a**) Measured vs. ideal values of test impedance magnitude, (**b**) phase. Diamonds: experimental data; dashed line: fitting.

First, several metal film resistors with nominal values ranging from 1 Ω to 1.2 kΩ were characterized with a precision LCR meter (E4980A Keysight, Santa Rosa, CA, USA) and then used as a load in the test of the AFE. The measured results are depicted in Figure 7a. A high *R*^2^ correlation coefficient exists between the resistance values obtained by means of the LCR meter (“ideal” values) and their counterparts measured by the bioimpedance sensor (“measured” values). For these measurements, a reference resistor *R_S_* of 500 Ω along with an excitation current having an amplitude of 120 µA and a frequency of 150 kHz, were used.

Once the bioimpedance sensor was experimentally evaluated over the whole specified impedance magnitude range, the measurement of the phase shift due to an *RC* test load was carried out. In this case, a resistor in the range from 1 Ω to 10 kΩ and a 50-nF capacitor connected in parallel were used as a load. Figure 7b shows the phase shift values obtained with the CMOS AFE (“measured” values) against the phase values provided by the *RC* test load measured with the LCR meter (“ideal” values). The same excitation current and the same reference resistor as in the preceding test were used. A high correlation between the measured and the ideal values (*R*^2^ = 0.9984) is again apparent.

**Figure 8:**
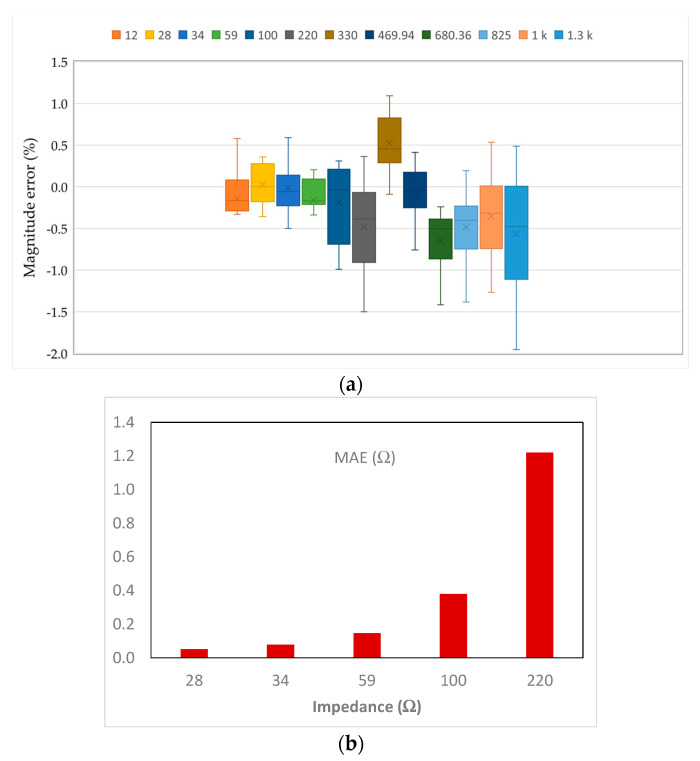
Statistical analysis of the measured impedance magnitude error in 10 test-chip prototypes: (**a**) Magnitude error (%) at 12 different resistor values; (**b**) MAE for the impedance measurement at TI range.

In order to validate the measurement capabilities of the proposed AFE for monitoring variations of human SV and CO, a number of measurements of impedance magnitude and phase were done in 10 AFE test-chip prototypes. Figure 8a presents the magnitude error at 12 different resistor values over the 12 Ω–1300 Ω range. The test results per magnitude value are represented by the box-and-whisker-plot, which illustrates that the measured error is smaller at intermediate magnitude values and the error is maximum at the upper limit.

In normal healthy subjects, the thoracic impedance (TI) is approximately 30 Ω. However, the normal value of TI varies between individuals, due to variations in chest size and morphology, and typically ranges from 20 Ω to 50 Ω [15]. Additionally, the electrode placement is a significant factor that contributes to TI measured value. In the literature, the basal TI value ranges from 20 Ω to 200 Ω. According to Figure 8a, for the target impedance interval, the measured error is below ±1%. Figure 8b shows the mean absolute error (MAE) in measuring the impedance magnitude for the intended TI range. The results are from 10 AFE test-chip prototypes and illustrate that it is possible to measure the impedance magnitude on average with less than 0.4 Ω error at each impedance value below or equal to 100 Ω, which can be considered sufficient for SV and CO assessment.

**Figure 9:**
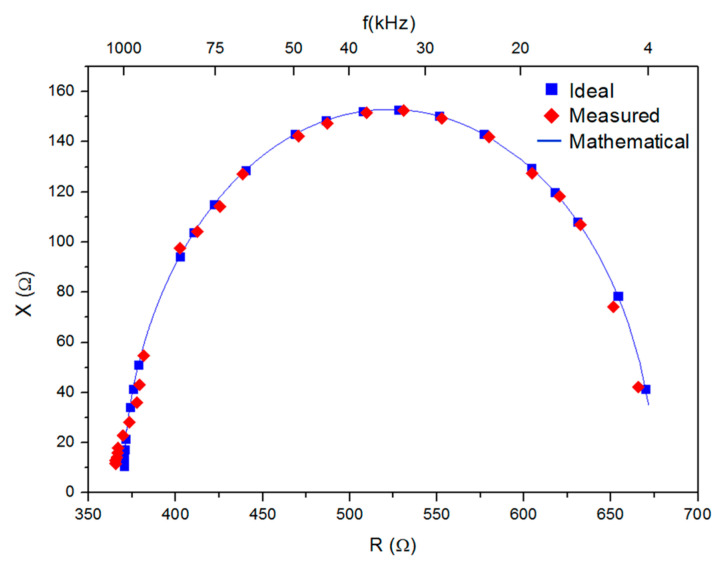
Cole–Cole plot in the impedance plane for the 2R1C circuit (*R_e_* = 681 Ω; *R_i_* = 909 Ω; *C_m_*/2 = 3.3 nF), measured by the bioimpedance sensor (red diamonds); ideal, i.e., obtained by the impedance expression using *R* and *C* values measured by the LCR meter (blue squares); and mathematical, i.e., obtained by the impedance expression using nominal *R* and *C* values (thin blue solid line).

Next, we conducted an experimental evaluation of the AFE behavior for in vivo measurements using the 2R1C equivalent circuit that was previously shown in Figure 1a. This *RC* series-parallel circuit combination consists of a 681 Ω resistor (*R_e_*) in parallel with the series connection of a 909 Ω resistor (*R_i_*) and a 3.3 nF capacitor (*C_m_*/2), selected to model a typical whole-body composition impedance response (one pair of electrodes placed in one hand and the other in one foot of the patient). Using the 2R1C equivalent circuit over a 4 kHz–1 MHz frequency interval allows characterizing the designed AFE under (emulated) in vivo conditions. Figure 9 compares the plot of the imaginary part, *X*, versus the real part, *R*, of the impedance of this circuit (Cole–Cole plot) measured by the bioimpedance device (“measured”), the plot (“ideal”) obtained by the theoretical impedance expression using the *R* and *C* values measured by the E4980A LCR meter, and the theoretical (i.e., “mathematical”) plot corresponding to the impedance expression using the nominal *R* and *C* values, for an excitation current amplitude of 200 µA. Very good agreement among the three plots is observed.

Table 1 summarizes the performance of the proposed AFE and compares it with the performance of other state-of-the art designs. Compared to the previous works, the proposed AFE achieves low power consumption while providing excellent magnitude and phase measurement accuracies at the same time.

### 4.2. Wireless Bioimpedance Device Experimental Performance

The experimental performance of the wireless bioimpedance device is summarized in Table 2. As stated before, the bioimpedance sensor has been designed to cover the whole bioimpedance variation range that takes place in the human body over the excitation frequency interval from 1 kHz to 1 MHz. Thus, the proposed wireless bioimpedance device can perform any type of local, regional, and whole-body bioimpedance analysis. The power consumption performance of the wireless device refers to two different states, i.e., the idle state, in which only the CMOS AFE is active, and the transmit and receive state, in which the whole circuitry for wireless communication is in active mode.

A wireless communication interface enables the user to control the device. More specifically, a graphical user interface facilitates its remote configuration as well as the analysis of the measured results in a remote server—PC, tablet, or smart phone. This software was developed in MATLAB, Phyton, and Android programming environments, thus opening the possibility of using the wireless device with Windows, OSX, and Android operating systems.

Two general possibilities exist for wirelessly configuring the bioimpedance device. One of them allows configuring the device to carry out an application which has been previously calibrated. In such cases, the operating parameters are stored in the smart device. The other possibility consists of using the device as a true measuring bioimpedance device where all operating parameters, such as the type of analysis—BIS or SF analysis—the amplitude of excitation current, and the frequency or the frequency interval and the number of frequencies in this interval, can be defined by the user.

Similarly, the graphical user interface also allows storing and displaying the values of the bioimpedance magnitude and phase measured by the wireless device. Additionally, the user can choose to perform various mathematical operations on the measured data for post-processing, or he/she can use a pre-defined strategy for a specific application.

### 4.3. Hemodynamic Assessment: A Monitor Patch for Cardiac Output

Hemodynamic monitoring has undergone significant advancements, and a number of minimally invasive and noninvasive methods for measuring SV and CO have been proposed since the introduction of the Swan–Ganz catheter more than 50 years ago [38]. Among noninvasive methods, variations of bioimpedance magnitude (ICG or TEB) in the thorax, e.g., thoracic impedance (TI), have been used to validate our bioimpedance sensing device for monitoring cardiac activity. SV calculation was carried out using (2) and following the indications recommended in [15].

**Figure 10:**
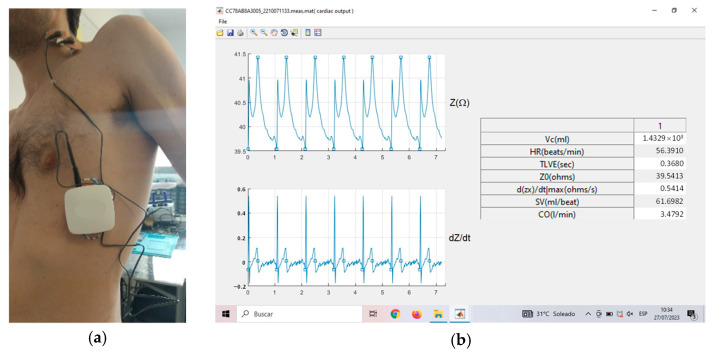
(**a**) Electrode placement for TI measurements in a healthy volunteer; (**b**) screen capture of the graphical user interface for SV and CO measurements.

To perform short-period TI measurements, the proposed bioimpedance device was configured to perform SF analysis with an excitation current frequency of 100 kHz. Unless specifically indicated otherwise, a tuning routine selects an adequate value of the amplitude excitation current according to the chosen reference resistor *R_S_* in order to adapt the measurement range to the value of the unknown mean basal TI at any moment. As shown in Figure 10a, the bioimpedance signal was obtained through two dual standard ECG spot electrodes. As stated in Section 2, one was connected above the base of the neck and below the ear and the other on the left side of the thorax (under the white enclosure), in the midaxillary line at the level of the xiphoid process. The two electrodes of the pair were placed close to each other on the neck, while the adjacent electrodes on the thorax were approximately 5 cm apart. The outer electrodes inject and collect, respectively, the excitation current, whereas the inner electrodes sense the response voltage signal, from which bioimpedance and ECG signals are obtained. Figure 10b illustrates the graphical user interface that is used to control the real-time data acquisition and visualization of bioimpedance in the thorax. This interface is used to track changes in SV and CO. 

**Figure 11:**
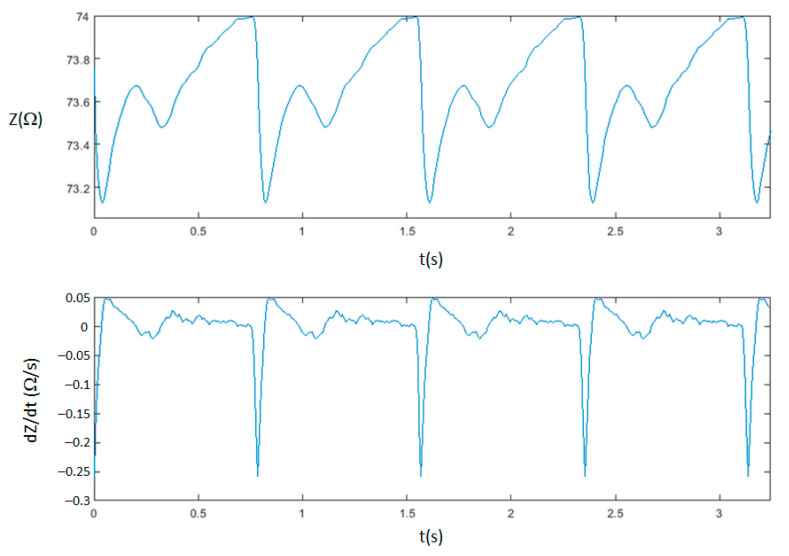
Pattern of *Z* and *dZ*/*dt* waveforms recorded by the proposed bioimpedance device.

To reduce breathing effects, the bioimpedance signals were averaged prior to determine (*dZ*/*dt*)_max_ and *T_LVE_*. A specific software routine running in the wireless bioimpedance device has been tailored to obtain SV from the changes occurring in the averaged bioimpedance magnitude waveform. Figure 11 illustrates the magnitude and the first derivative of the bioimpedance signal recorded by the proposed wireless bioimpedance device on four consecutive cycles. Since the morphology of these signals is not stationary, the recordings are preprocessed with averaging, detrending, and filtering functions. Moreover, intra- and inter-patient morphological variability of the waveforms is the major trouble for the detection of the characteristic points of the *dZ*/*dt*, especially for older patients who suffer from arterial stiffness [39]. Therefore, a data-driven algorithm customized to the expected characteristic points, i.e., BCXYO complexes, as a consequence of the physiological changes in the vascular system, was developed for robust recognition of the *T_LVE_*.

**Figure 12:**
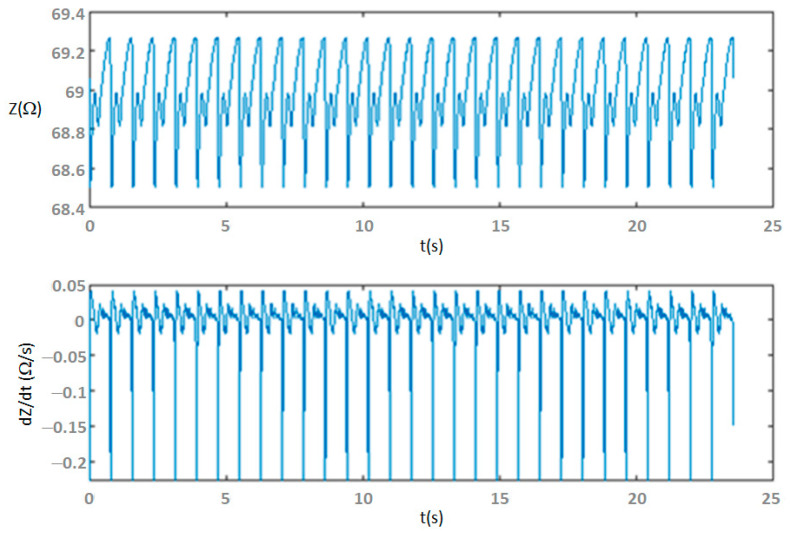
Thoracic impedance signals (*Z* and *dZ*/*dt*) during cardiac output monitoring test.

The functionality of the wireless bioimpedance device for CO monitoring was clinically validated against Doppler echocardiography as a reference method. To this end, the SV was simultaneously measured with commercial Doppler echocardiograph equipment and the proposed bioimpedance device, in a total of 33 patients with different heart diseases, ages (≥55), and genders. Clinical tests were carried out at the Cardiology Service of the Hospital Universitario de Badajoz (Spain). As an example, Figure 12 illustrates TI signals of one of the cardiac patients, and the corresponding results are given in Table 3.

**Figure 13:**
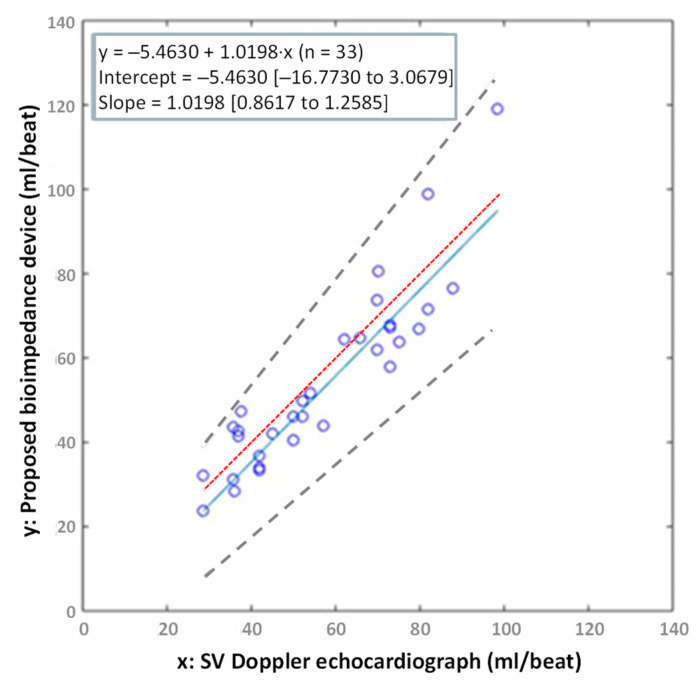
Passing–Bablok plot of correlation of SV measured with the proposed bioimpedance device and a Doppler echocardiograph [*R*^2^ = 0.8308]. The plot shows the observations with the regression line (solid blue line), the confidence interval for the regression line (dashed lines) and identity line (x = y, dotted red line).

A suitable technique for comparing two different methods/instruments—Passing–Bablok procedure [40,41]—was applied to the two sets of measured results obtained with the Doppler echocardiograph and the proposed wireless bioimpedance sensing device. Figure 13 corresponds to the correlation plot of SV results. The straight line of best fit is plotted as a solid line (blue trace) together with the upper and lower 95% confidence intervals, which are represented as dashed lines, and the line of identity, which is shown as a dotted line (light red trace). The inset in Figure 13 includes the Passing–Bablok regression formula (first row) and the two 95% confidence limits on intercept and slope (second and third row, respectively). From these results, we can conclude that the two methods are substantially equivalent (*R*^2^ *=* 0.8308).

## 5. Discussion

The purpose of this work was to design a wireless bioimpedance-based device and to explore its potential as a clinical IoMT monitor for noninvasive and continuous hemodynamic assessment. It is the first part of an ambitious project that aims to develop a multi-modal and wearable device capable of continuously assessing hemodynamics and various physiological parameters related to breath behavior, pulmonary edema, and muscular physiology. The development of a medical prototype with these characteristics faces several challenges. These challenges include the need to balance processing requirements with power consumption, the use of non-allergenic materials, which are also very robust and have high endurance to sustain continuous cleaning, and the need to remain safe for the user even in single-fault conditions as specified in the IEC60601-1. As a result, we sought the advice of the Cardiology Service at the University Hospital (Badajoz, Spain) at the start of our project. They kindly provided us valuable assistance in the ergonomic and functional design of the prototype, as well as the information (bioimpedance data) that our device needs to provide them. This was the starting point to establish the requirements that the bioimpedance-based device needed to guarantee, which led to the need to design, manufacture, and test our own sensor instead of using a commercially available one. Then, the transistor-level design phase of the integrated circuits and their shipment to manufacturing started. From the moment that sensor prototypes were sent to manufacturing, the testing platforms to verify their performance began to be planned. Printed circuit board design techniques were applied to ensure a high degree of noise immunity so as not to reduce the dynamic range of the sensor. With respect to the whole device, appropriate infrastructure was implemented for TRL4 trials as well as to validate BLE connectivity to the app and server platform to report the results. Thanks to the collaboration between our research group and industry, the first prototype was achieved in a short time, considering the complexity of the reported activities.

### 5.1. Device Upgrades

We have shown that our device can capture high-quality hemodynamic parameters. However, the packaging solution is not ideal for extended measurements, particularly for patients who are lying in bed. The resulting device prototype uses a housing that is slightly larger than necessary for the discrete electronics. However, there is a straightforward upgrade that can be made to improve its size and usability. For instance, the AC current generator can be implemented as a CMOS integrated circuit, which will maintain the proper bandwidth, and be packaged with the already tested AFE. This refinement will also reduce the total power consumption, resulting in improved battery life. Our next steps will involve integrating the bioimpedance device into a garment that has textile electrodes. We will also add an algorithm to reduce artifacts [42], which will improve the measurement of bioimpedance signals.

### 5.2. Study Limitations

Regarding medical device development, this study should be seen as a demonstration of the device’s feasibility, i.e., proof-of-concept, and safety in real-life conditions (TRL4). The small number of patients (n = 33) was chosen based on the hospital’s workflow and the limited time available to collect continuous data. Additionally, the validation was only conducted with patients who had the CHF condition. Once the proposed device upgrades are finished, we will conduct a new study with a larger number of patients to evaluate the device’s performance more accurately.

## 6. Conclusions

The proposed wireless bioimpedance-based device consists of four major functional units: a custom-made CMOS AFE integrated circuit for a bioimpedance sensor, a digitally programmable AC current generator, a low-power microcontroller unit with integrated ADC and RF transceiver section, and a power management unit. The CMOS AFE was designed using a single-ended polar demodulator architecture, based on the MRPDD method, which allows to achieve a bandwidth up to 1 MHz and to efficiently minimize the effect from several error sources.

The proposed bioimpedance device has been designed and fabricated to be incorporated into a multi-modal and wearable device, which combines both continuous hemodynamic assessment and physiological parameters related to breath behavior, pulmonary edema, and muscular physiology measuring capabilities. According to the experimental results, the measurement ranges for magnitude and phase are 1 Ω–1.2 kΩ and 5°–80°, respectively. These measurement ranges have a resolution of 12 bits. As a target IoMT application, a cardiac activity monitor was experimentally evaluated in this work. Currently, the clinical data show that the presented device is substantially equivalent to a Doppler echocardiograph for stroke volume and cardiac output monitoring.

## Figures and Tables

**Table 1:** **P**erformance summary and comparison with state-of-the-art works.

Parameters	[31]	[32] *	[33]	This Work
Measurement Method	Simplified MRPDD	Polar demodulation	Polar demodulation	MRPDD
CMOS process (μm)	0.35	0.25	0.18	0.35
Supply Voltage (V)	±2.5	2.5	1.8	2
Current Consumption	4 mA ^(1)^	N/A	N/A	0.75 mA ^(1)^
Power consumption	21 mW	<10.3 mW	0.756 mW	1.5 mW
Frequency Range (Hz)	100–100 k	1 k–2 M	100–10 M	1 k–1 M
Measurement Range	≤5 kΩ, ≤70°	N/A	≤7 kΩ, ≤70°	≤1.2 kΩ ^(2)^, ≤80°
Accuracy Error	Mag.: <3.5% Phase: <3.6°	Mag.: <1% Phase: <1.3°	Mag.: <1.1% Phase: <1.9°	Mag.: <1.2% Phase: <1.5%
Active area (mm^2^)	0.4	0.48	1.95	0.3
FoM (kHz/mW/area)	11.9	404	6783	2222

* Simulation results. ^(1)^ Power consumption of the excitation current source is not included. ^(2)^ Extended range up to 2.7 kΩ.

**Table 2:** Experimental performance of the wireless bioimpedance device.

Measuring technique	Tetra-polar/MRPDD	
Bioimpedance analysis types	Time domain [ZX(t)]	
	Spectroscopy [ZX(ω)]	
Excitation current (Sinusoidal)	Amplitude	5 µA–1 mA
	Frequency	1 kHz–1 MHz
Measurement ranges	Magnitude	1 Ω–1.2 kΩ
	Phase angle	5°–80°
Resolution	12 b (10-bit ENOB)	
Communication protocol	Bluetooth Low Energy 4.0	
Power supply	3-V single supply	
Power consumption	1.65 mW (idle mode)	
	27 mW (transmit/receive mode)	

**Table 3:** Experimental Hemodynamical Assessment.

Noninvasive Method	HR (beat/min)	SV (mL/beat)	CO (mL/min)
Doppler Echocardiography	79	52.5	4.15
Proposed Bioimpedance Device	76.5	52.9	4.05

## Data Availability

Not applicable.
